# Multimodality treatment for brain arteriovenous malformation in Mainland China: design, rationale, and baseline patient characteristics of a nationwide multicenter prospective registry

**DOI:** 10.1186/s41016-022-00296-y

**Published:** 2022-10-17

**Authors:** Yu Chen, Heze Han, Li Ma, Ruinan Li, Zhipeng Li, Debin Yan, Haibin Zhang, Kexin Yuan, Ke Wang, Yang Zhao, Yukun Zhang, Weitao Jin, Runting Li, Fa Lin, Xiangyu Meng, Qiang Hao, Hao Wang, Xun Ye, Shuai Kang, Hengwei Jin, Youxiang Li, Dezhi Gao, Shibin Sun, Ali Liu, Shuo Wang, Xiaolin Chen, Yuanli Zhao

**Affiliations:** 1 Department of Neurosurgery, Beijing Tiantan Hospital, Capital Medical University, Beijing, China; 2 Department of Neurosurgery, Peking University International Hospital, Peking University, Beijing, China; 3 Department of Interventional Neuroradiology, Beijing Tiantan Hospital, Capital Medical University, Beijing, China; 4 Department of Gamma-Knife Center, Beijing Tiantan Hospital, Capital Medical University, Beijing, China; 5 China National Clinical Research Center for Neurological Diseases, Beijing, China; 6 Stroke Center, Beijing Institute for Brain Disorders, Beijing, China

**Keywords:** Arteriovenous malformation, Embolization, Microsurgery, Multidisciplinary assessment, Outcomes, Radiosurgery, Registry, Rupture

## Abstract

**Background:**

Brain arteriovenous malformation (AVM) is an important cause of hemorrhagic stroke in young adults, which can lead to severe neurological impairment. The registry of Multimodality treatment for brain ArTeriovenous malformation in mainland CHina (MATCH) is a national prospective registry to identify the natural history of AVMs in Asian population; to investigate traditional and emerging hemorrhagic predictors; and to explore the superiority of the multidisciplinary assessment in improving the long-term outcomes.

**Methods:**

Consecutive AVM patients will be enrolled from 52 participating hospitals in mainland China. Baseline demographic, clinical and imaging data will be collected prospectively. Conservation, microsurgery, embolization, stereotactic radiosurgery (SRS), and multimodal strategies are all included in this study. Patients will be divided into experimental and control group according to whether the treatment protocols are formulated by multidisciplinary team. Neurofunctional status, subsequent hemorrhage, seizure, and novel neurofunctional deficit will be queried at 3 months, annually (1 and 2 years), 3 years, and 10 years follow-up.

**Results:**

Between August 2011 and April 2021, 3241 AVMs were enrolled in 11 participating sites. Among them, 59.0% were male with an average age of 28.4 ± 14.6 years, 61.2% had rupture history and 2268 hemorrhagic events occurred before admission. The median Spetzler-Martin grade and Lawton-Young grade was 3 and 5, respectively. Microsurgery is the dominant strategy (35.7%), with a similar proportion of embolization, SRS, and a combination of both (12.7%; 14.8%; 11.8%; respectively). Among them, 15.43% underwent multidisciplinary assessment and received standardized treatment. At the most recent follow-up, 7.8% were lost and the median follow-up duration was 5.6 years.

**Conclusions:**

The MATCH study is a large-sample nationwide prospective registry to investigate multimodality management strategy for AVMs. Data from this registry may also provide the opportunity for individualized risk assessment and the development of optimal individual management strategies.

**Trial registration:**

ClinicalTrials.gov Registry (NCT04572568).

**Supplementary Information:**

The online version contains supplementary material available at 10.1186/s41016-022-00296-y.

## Background

Brain arteriovenous malformations (AVMs) are tangles of abnormally dilated vessels without intervening capillaries, which represent high-flow and low-resistance hemodynamic features due to direct arteriovenous shunting [1]. AVMs usually manifest as intracranial hemorrhage (30–70%), seizure (10–30%), headache, or incidental findings (0–15%) [2]. The natural annualized rupture risk of AVMs was estimated to 1–3% per year if left untreated [3]. Non-White race was suggested as an independent hemorrhagic factor in a widely accepted prediction model [4]. However, the finding have not been confirmed in the predominantly yellow Asian population, and some scholars have expressed concerns about it [5]. Therefore, clarifying the natural history of AVMs among Asian populations is of distinct practical significance [6].

The primary purpose of treatment in AVMs is to avoid neurological impairment due to future hemorrhagic stroke [7]. The scientific statement of AHA/ASA proposed that the definitive treatment of AVMs should be complete elimination of the nidus and the arteriovenous shunt [8]. Three interventional modalities have been developed: microsurgery, embolization, and stereotactic radiosurgery (SRS). The Spetzler-Martin (SM) grading system is widely used to estimate the risk of morbidity and mortality attending the operative treatment [9]. Generally, SM I/II AVMs are amenable to microsurgery alone. Embolization and SRS also have been indicated can achieve favorable outcomes with limited morbidity and mortality. SM III AVMs typically require multimodal approach. SM IV/V AVMs must be treated conservatively unless ruptured [10]. In 2014 and 2020, A Randomized Trial of Unruptured Brain Arteriovenous Malformations (ARUBA) concluded that the natural history of unruptured AVMs is better than any form of treatment [11, 12]. Despite the enormous controversy, this result still significantly altered the treatment decision for AVMs [13]. However, conservation of unruptured AVMs assessed as high rupture risk might not be justified. Since the neurological impairment caused by AVM rupture can be fatal, individualized rupture and prognostic prediction models are required to determine whether to take interventions. A recently released expert consensus on the management of AVMs recommends that a multidisciplinary professional committee of neurosurgeons specializing in AVM resection, embolization, and radiation conduct multimodal assessments and create individualized treatment strategies [10].

We conducted this study to identify the natural history of AVMs in the Asian population, investigate the traditional and emerging hemorrhagic predictors, and explore the superiority of the multidisciplinary assessment in improving the long-term outcomes. In this report, we introduced the design, rationale, baseline data, as well as the strengths and potential limitations of the study.

## Methods

### Overview of the MATCH study

**Fig. 1:**
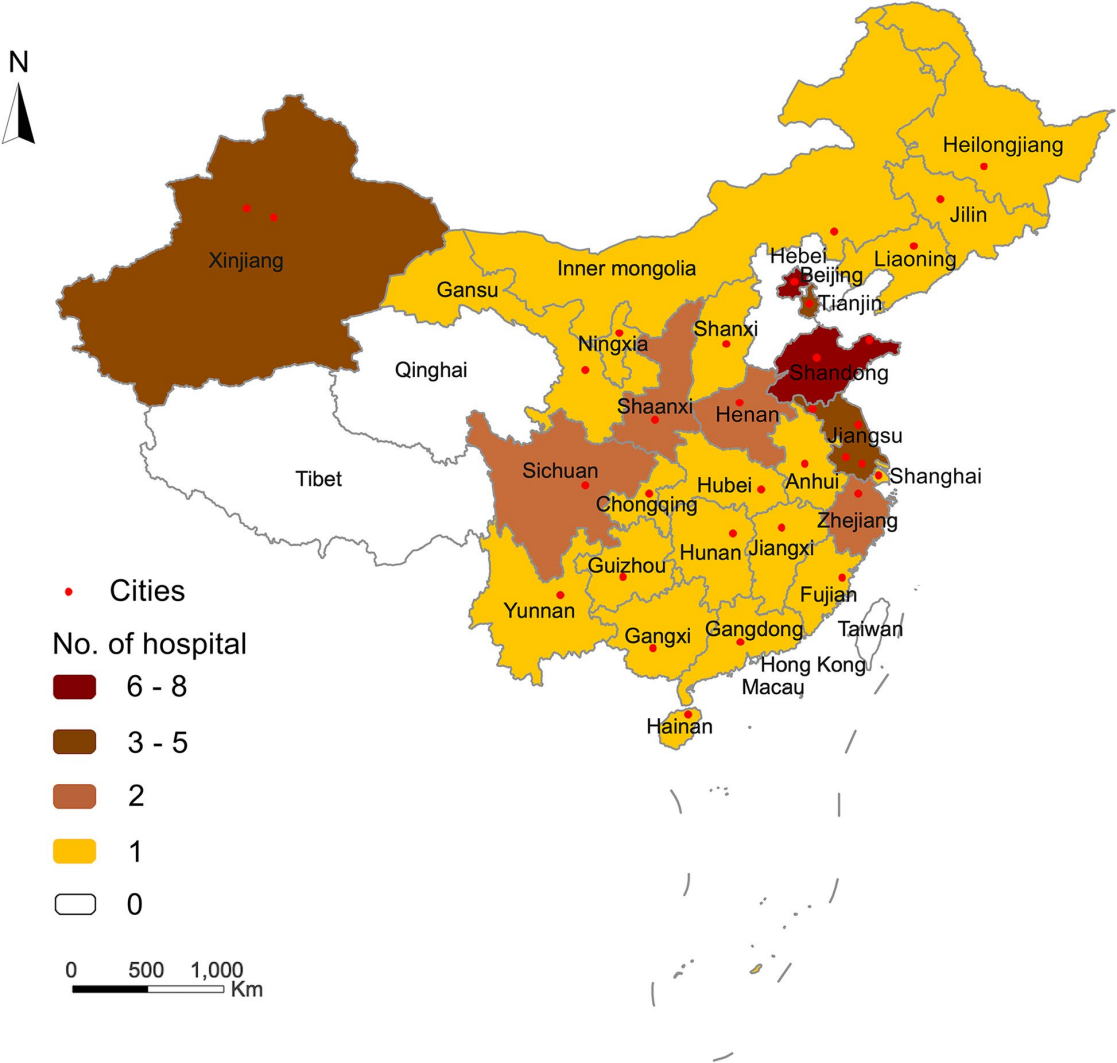
The geographical locations of participating sites in MATCH

The registry of Multimodality treatment for brain ArTeriovenous malformation in mainland CHina (MATCH) is a national multicenter prospective registry that recruited consecutive AVMs from 52 tertiary hospitals that cover 20 provinces, 4 ethnic minority autonomous regions, and 4 municipalities in mainland China. An organizing committee composed of members from Beijing Tiantan Hospital and several neurosurgical centers prepared the design and organization. About 100 tertiary hospitals (about 4 in each province) that can cover most of the population in mainland China passed the initial screening by the MATCH Research Organizing Committee. About 60 hospitals were initially invited, and 52 hospitals were finally identified, which declared that they had reliable research capabilities and were willing to devote themselves to this study. A full list of MATCH study members can be found in Supplementary material 1, and Fig. 1 shows the geographical location of all participating hospitals.

The main objectives of this study are to identify the natural history of AVMs in the Asian population, investigate traditional and emerging hemorrhagic predictors, and explore the superiority of the multidisciplinary assessment in improving the long-term outcomes. Individuals who complete baseline demographic, clinical, and imaging data collection will be included in the prospective cohort and will be followed up for at least 3 years. This study will last for 20 years, and cross-sectional and longitudinal designs will be used for data analysis on different research topics.

### Study objectives

The objectives of the MATCH study were to identify the natural history of AVMs in the Asian population; to investigate the traditional and emerging angioarchitecture, hemodynamic, genetic, and environmental risk factors associated with AVM rupture; and to explore the superiority of the multidisciplinary assessment in improving the long-term outcomes. Based on this, individualized rupture and prognostic prediction models will be established to promote the precision medicine.

### Study design

This study is a national multicenter prospective registry study. First, the MATCH study will analyze the natural history of AVMs in the Asian population through a Real-World study, explore the relevant factors affecting long-term outcomes through case–control studies, and establish prognostic predictive models. This study will embed nested case-controlled (NCC) studies to explore traditional as well as novel hemorrhagic predictors, and establish reliable predictive models for AVM rupture. The MATCH study will also analyze the superiority of the multidisciplinary assessment in improving the long-term outcomes of AVMs through cohort studies.

### Patient enrollment

All AVMs diagnosed at participating hospitals will be candidates for this study, which is expected to include approximately 4000 AVMs between August 1, 2011 and April 1, 2032. The inclusion criteria are as follows: (1) the diagnosis of AVM is confirmed with digital subtraction angiography (DSA) and/or magnetic resonance imaging (MRI); (2) patients with complete clinical and imaging data; (3) patients or their guardians agree to collect personal information for this study and sign informed consent. The exclusion criteria are as follows: (1) patients undergo intervention management before admission, such as microsurgery, embolization, or SRS; (2) patients diagnosed with spinal AVMs; (3) patients missing critical baseline demographic, clinical, and imaging data.

### Baseline data collection

The data collection is performed through face-to-face interviews and examinations by centralized trained personnel following a standard data collection protocol developed by the MATCH Research Organizing Committee (Table 1). The information including demographics, medication history, neurological status, laboratory tests, imaging characteristics, and hemodynamic parameters.

**Table 1:** Schedule of evaluation in the MATCH study

Characteristics	Screening and Entry	Pre-treatment	Discharge	3 months follow-up	Annual follow-up (1 and 2 years)	3 years follow-up	10 years follow-up
Diagnosis	X						
Written informed consent	X						
Past treatment history	X						
Demographic data	X	X					
Medical history	X	X		X	X	X	X
Medication history		X	X	X	X	X	X
Physical examination	X	X	X	X	X	X	X
Clinical neurofunctional assessment	X	X	X	X	X	X	X
CTA scanning	X	X	X				
MRI scanning	X	X		X	X	X	X
DSA examination	X	X		X		X	X
Hemodynamic evaluation		X		X		X	X
Treatment strategy			X				
Complications			X	X	X	X	X
Obliteration rate			X	X	X	X	X
Hemorrhagic vascular events			X	X	X	X	X

*CTA* Computed Tomography Angiography, *DSA* Digital Substraction Angiography, *MATCH* Registry of Multimodality treatment for brain ArTeriovenous malformation in mainland China, *MRI* Magnetic Resonance Imaging

An electronic data capture (EDC) system has been developed and used for data collection. The patient can be registered via a web-based system that will structure the forgoing characteristics (https://artt.neurochina.com). The EDC system is available 24 h a day and can automatically check the integrity and logical error of the uploaded data. An independent contract research organization will performs regular independent data monitoring through the EDC to troubleshoot and question data where errors exist. All data are anonymized prior to data analysis.

### Demographic and clinical characteristics

The demographic data included sex, ethnicity, admission age, family history, diet, physical activity, educational attainment, lifestyle, smoking status, drinking status, and medication history.

The clinical characteristics included onset manifestation (hemorrhage, seizure, headache, neurofunctional deficit, accidentally discovered, others), admission modified Rankin Scale (mRS), time of each rupture, treatment modalities (conservation, microsurgery, embolization, SRS, multimodality strategy, etc.), discharge obliteration rate, perioperative complications (hemorrhage, seizure, intracranial infection, etc.), discharge mRS, etc. The hemorrhagic presentation is defined as hemorrhage attributable to AVM rupture with symptomatic and CT evidence. Seizure is classified as generalized and partial. The evaluation of mRS was conducted by trained who have at least 5 years of experience in clinical practice.

### Imaging data collection

MRI and DSA are recommended for all AVMs in the MATCH study. For MRI, three-dimensional T1-weighted magnetization prepared rapid acquisition gradient echo (3D T1w MPRAGE), two-dimensional T2-weighted (2D T2w), susceptibility-weighted imaging (SWI), three-dimensional time-of-flight MR angiography (3D-TOF MRA) are included as mandatory sequences at baseline, 3-month, annually (1 and 2 years), 3-year, and 10-year follow-up. Resting-state fMRI and diffusion tensor imaging (DTI) are the recommended sequences for AVMs involving eloquent areas for treatment decision. DSA examination is recommended when evaluating the rupture risk, choosing a treatment strategy, and determining the postoperative obliteration rate. Besides, 3-dimensional DSA is considered necessary if a combined aneurysm is suspected. In terms of hemodynamic assessment, the MATCH study uses color-coded DSA post-processing technique (syngo iFlow, Siemens, Berlin, Germany) to obtain time-dependent hemodynamic parameters [14]. Trained investigators will conduct all the MRI and DSA based on standardized protocol. These imaging techniques enable us to observe angioarchitecture features, screen hemorrhagic predictors, monitor the progression, and assess the risk of secondary hemorrhage. Imaging data were collected in the form of digital images and communications in the form of medical CDs (DICOM format) and analyzed by the Imaging Committee (a multidisciplinary team of Neurosurgery, Interventional Neuroradiology, Radiosurgery, and Imaging Research Center) of the MATCH Research Organizing Committee.

The definition of angiographic characteristics is consistent with the reported terminology provided by the joint committee led by the American Society of Interventional and Therapeutic Neuroradiology [15]. The hemodynamic parameters included time to peak (TTP), inflow gradient, outflow gradient, stasis index, full width at half maximum (FWHM), trans-nidal relative velocity (TRV), etc. [16, 17]. All the angioarchitecture and hemodynamic parameters are independently interpreted manually and double-blind. If there is doubt about interpretation, the Imaging Committee will arbitrate and analyze.

### Clinical management

Conservation, microsurgery, embolization, SRS, and multimodal strategies are all included in this study. Patients will be divided into experimental and control groups according to whether the treatment protocols were formulated by a multidisciplinary team. In order to reduce workload, the MATCH Research Organizing Committee developed a standardized treatment protocol of multidisciplinary assessment as follows:

Ruptured AVMs: (1) microsurgery: microsurgery is recommended for AVMs not located in deep critical eloquent areas (brainstem, thalamus, basal ganglia, etc.) or more than 5 mm away from functional fiber bundles, regardless of the SM grade; (2) embolization: embolization should be performed 2–6 weeks after rupture; target embolization for hemorrhagic predictors could be considered as a monotherapy; palliative embolization can be used as an adjunctive strategy to reduce blood flow or nidus volume before microsurgery or stereotactic SRS; (3) SRS: SRS is indicated for patients with a nidus volume < 10 ml and not in the acute phase of hematoma (< 3 months); volume-stage or dose-stage can be used for giant AVMs. (4) Multimodality: the multimodality strategy such as single-stage combined embolization + resection (hybrid surgery) or staged embolization + resection or adjunctive embolization + resection/SRS or adjuvant SRS + resection are indicated for complex AVMs, such as giant AVMs involving deep eloquent areas, or AVMs with complex blood supply. (5) Conservation: conservation can be used for AVMs that are prone to severe disability due to intervention.

Unruptured AVMs: interventions are recommended if unruptured AVMs are assessed as being at high rupture risk or having refractory epilepsy or acceptable postoperative neurological deficits; otherwise, conservative treatment is recommended. The rupture risk stratification will be assessed qualitatively and quantitatively by the multidisciplinary team based on their own experience and the rupture prediction model to facilitate the division of the experimental and control groups. Regular multidisciplinary discussion meetings and fixed multidisciplinary groupings will ensure consistency of analytical results. Besides, the choice of intervention is the same as for ruptured AVMs.

It should be noted that the AVM multidisciplinary team for the MATCH study was established in June 2018, so the previously collected prospective AVM cohort from August 2011 to June 2018 and the AVM cohort after June 2018 without comprehensive evaluation by the multidisciplinary team served as the control group.

### Follow-up assessment

Neurofunctional status (mRS), subsequent hemorrhage, seizure, headache, novel neurofunctional deficit, and medication were queried at 3 months, annually (1 and 2 years), 3 years, and 10 years follow-up by trained research coordinators. Medical records and imaging examination data will be collected for suspected individuals with cerebral vascular events. An independent final review committee will assess suspected vascular events without hospitalization.

The MATCH study will conduct routine imaging follow-up by MRI, which is consistent with that described above. Due to the dose-cumulative effect, we will use DSA and MRI at the third year to determine the long-term obliteration rate, regardless of the intervention strategies. We will use imaging at 10th year after surgery to assess the ultra-long-term imaging prognosis. Color-coded DSA (follow-up DSA) was used to assess short-term and long-term time-dependent hemodynamic changes in residual nidus, peri-nidus, and cerebral hemispheres after treatment.

### Outcomes measurements

The primary outcome: neurofunctional status (mRS score) at 3 years after treatment. The secondary outcomes: obliteration rate, subsequent hemorrhage, complication rate, and improvement in clinical symptoms (epilepsy, headache, neurological dysfunction) at 3 years after treatment. The obliteration rate will be evaluated by DSA or MRI, as several previous studies have suggested that MRI could provide a similar assessment of the obliteration rate as DSA [18]. The subsequent hemorrhage is defined as intracranial hemorrhage that could be attributed to AVM rupture with symptomatic and CT evidence. Postoperative complication included novel neurological dysfunction, radiation-related complications, etc. The prognosis of epilepsy will be assessed by Engle classification. Post-treatment headache will be assessed by the WHO pain grading classification.

### Statistical analysis

Categorical variables were presented as percentages and continuous variables as mean with standard deviation (SD) or median with interquartile range (IQR). In univariable analyses, *t* test or Mann–Whitney test will be used to compare continuous variables, whereas *χ*2 test or Fisher’s exact test will be used to compare the categorical variables. Multivariable logistic regression will be used to evaluate the predictors of hemorrhage and long-term neurological outcomes in the cross-sectional dataset, and ORs with their 95% CIs will be evaluated. Univariable and multivariable Cox proportional hazard regression model or competing risk analysis or NCC will be performed to explore the predictors of subsequent hemorrhage in the survival dataset, and HRs with their 95% CIs will be evaluated. Kaplan–Meier plots of time-to-event outcomes of subsequent hemorrhage or complete obliteration (for AVMs who underwent SRS) among different treatment modalities will be presented. Generalized linear models or generalized linear mixed models will be used to explore potential correlates for continuous dependent prognostic parameters. Receiver operating curves (ROCs) will be used to assess the predictive validity in constructing predictive models for subsequent hemorrhage or long-term neurological outcomes. In cohort studies, propensity score matching (PSM) and inverse probability of treatment weighted (IPTW) will be used to adjust for confounding factors. Statistical significance was set at *p* < 0.05 (two-sided).

In this article, we compared the baseline demographic, clinical, and angioarchitecture characteristics of the multi-disciplinary and single-disciplinary assessment groups. Besides, although quality control has been maximized through the EDC system, there are still some missing values (less than 2%), which we have also reported. Proportions were used to describe the categorical variables, and means with SD or median with the IQR were used for continuous variables. Statistical analysis was performed using SPSS (version 25.0, IBM, New York, USA).

## Results

**Fig. 2:**
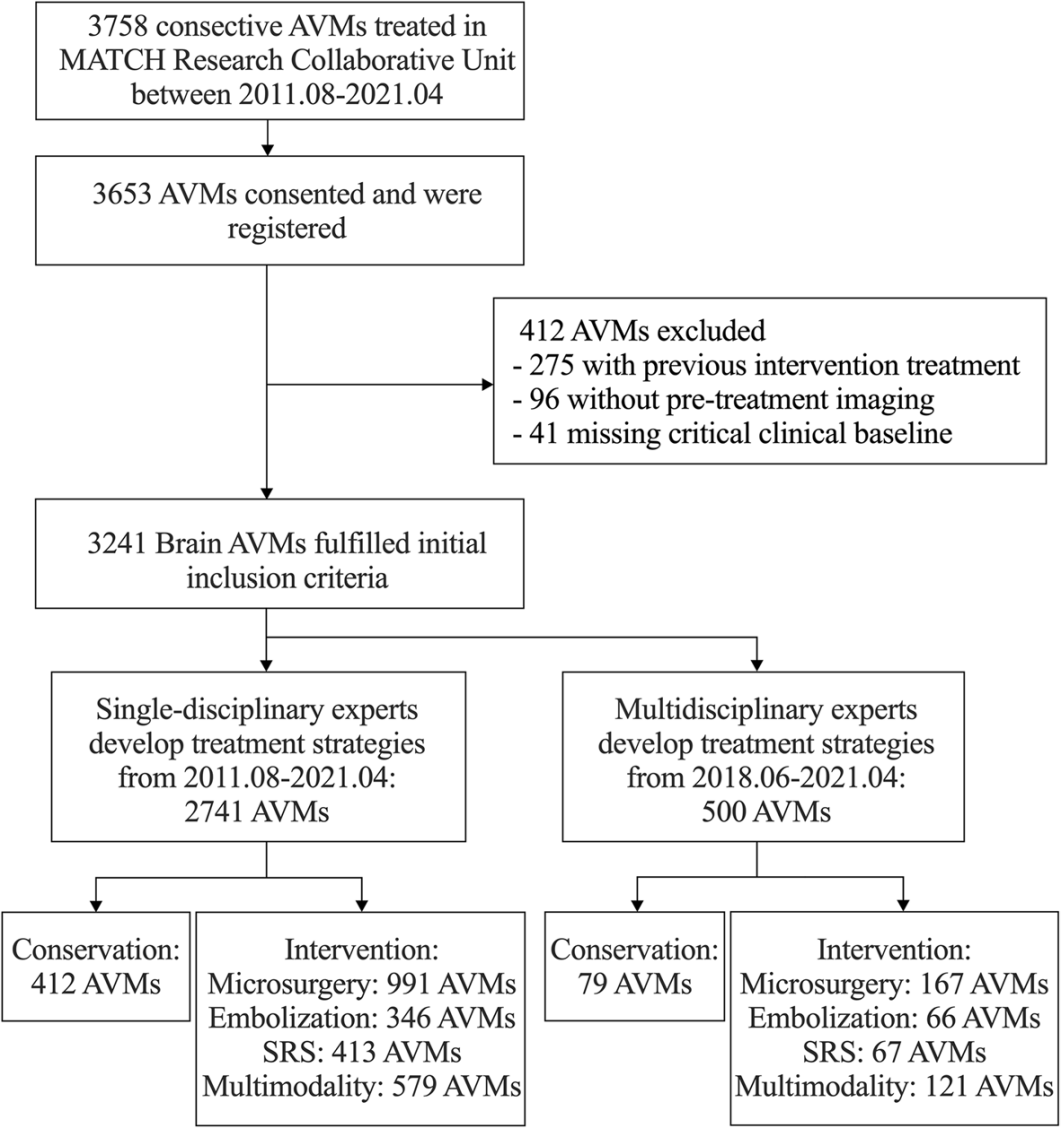
Flow chart of patients’ enrollment in MATCH. AVM, arteriovenous malformation; MATCH, registry of multimodality treatment for brain arteriovenous malformation in mainland China; SRS, stereotactic radiosurgery

Patient recruitment is still ongoing, and we now summarize the data in stages as follows: Between August 2011 and April 2021, 3653 brain AVM patients were consented and registered to the MATCH study in 11 participating sites. After rigorous screening, a total of 412 patients were excluded. Finally, a total of 3241 AVMs fulfilled the initial inclusion criteria. Among them, 15.43% (500 AVMs) underwent multidisciplinary assessment and received standardized treatment as prescribed by MATCH. The detailed patient enrollment flow chart is shown in Fig. 2. The included patients had similar baseline demographic, clinical, and imaging characteristics as those excluded (Table 2).

**Table 2:** Baseline characteristics of included and excluded AVMs in the MATCH study

Characteristics	Included	Excluded	*P* value
Baseline demographic data	*N* = 3241	*N* = 412	
Sex, male, n (%)	1911 (59.0)	240 (58.3)	0.782
Age (years)			
Mean ± SD	28.4 ± 14.6	28.3 ± 12.2	0.831
Baseline clinical data^a^	*N* = 3241	*N* = 371	
Onset manifestation			
Hemorrhage, n (%)	1984 (61.2)	226 (60.9)	0.911
Seizure, n (%)	754 (23.3)	84 (22.6)	0.858
Admission mRS			
Median, IQR	1 (1, 2)	1 (0, 2)	0.788
Baseline imaging data^b^	*N* = 3241	*N* = 316	
Location, supratentorial, n (%)	2844 (87.7)	281 (88.9)	0.542
AVM maximum diameter (cm)			
Mean ± SD	3.4 ± 1.9	3.3 ± 2.0	0.857
Eloquence, n (%)	1794 (55.3)	180 (56.9)	0.583
Deep venous drainage, n (%)	1284 (39.6)	122 (38.6)	0.726
Spetzler-Martin Grade			0.848
I-III	2694 (83.1)	264 (83.5)	
IV-V	547 (16.9)	52 (16.5)	

*AVM* Arteriovenous Malformation, *IQR* Interquartile Range, *N* Denominator, *n* numerator, *MATCH* Registry of Multimodality treatment for brain ArTeriovenous malformation in mainland China, *mRS* Modified Rankin Scale, *SD* Standard Deviation

^a^ 41 AVMs in the excluded cohort missing critical baseline clinical data

^b^ 96 AVMs in the excluded cohort missing critical baseline imaging data

Baseline demographic and clinical characteristics of the included AVMs are presented in Table 3. Overall, 59.0% were male, and the average age was 28.4 years. Among all enrolled patients, 61.2% (1984 cases) had rupture history, and 2268 rupture events occurred before admission. The median duration from diagnosis to treatment (including intervention and conservation) was 2.9 months. Microsurgery is the dominant treatment strategy (35.7%), 12.7% received endovascular embolization as monotherapy, and 14.8% underwent SRS. Staged embolization + SRS is the most common multimodal treatment strategy (11.8%). At the most recent follow-up, 7.8% were lost. The median follow-up duration was 5.6 years, and 79.4% have completed 3 years of follow-up. There were no statistically significant differences in all demographic and clinical characteristics between single-discipline and multidiscipline cohorts except for the follow-up duration.

**Table 3:** Baseline demographic and clinical characteristics of the participants in the MATCH study

Characteristics	All patients	Single-disciplinary cohort (2011.08–2021.04)	Multidisciplinary cohort (2018.06–2021.04)	*P* value
No. of patients	*N* = 3241	*N* = 2741	*N* = 500	
Ethnicity, male, n (%)	1911 (59.0)	1624 (59.2)	287 (57.4)	0.440
Age (years), mean ± SD	28.4 ± 14.6	28.3 ± 14.4	29.4 ± 15.6	0.118
Race, Han, n (%)	3109 (95.9)	2629 (95.9)	480 (96.0)	0.929
Onset manifestation				
Hemorrhage, n (%)	1984 (61.2)	1677 (61.1)	307 (61.4)	0.927
Seizure, n (%)	754 (23.3)	647 (23.6)	107 (21.4)	0.283
Headache, n (%)	698 (21.5)	587 (21.4)	111 (22.2)	0.695
Neurofunctional deficit, n (%)	583 (18.0)	488 (17.8)	95 (19.0)	0.522
Others, n (%)	271 (8.4)	230 (8.4)	42 (8.4)	0.995
Frequency of hemorrhage before admission, n	2268	1917	351	0.906
Type of seizure, generalized, n (%)	629 (19.3)	542 (19.7)	87 (17.4)	0.217
Duration from diagnosis to treatment (months), median, IQR	2.9 (0.9, 12.8)	2.8 (0.9, 12.9)	3.0 (0.9, 12.6)	0.471
Admission mRS, median, IQR	1 (1, 2)	1 (1, 2)	1 (1, 2)	0.653
Type of treatment				
Conservation, n (%)	491 (15.1)	412 (15.0)	79 (15.8)	0.659
Microsurgery, n (%)	1158 (35.7)	991 (36.2)	167 (33.4)	0.237
Embolization, n (%)	412 (12.7)	346 (12.6)	66 (13.2)	0.722
SRS, n (%)	480 (14.8)	413 (15.1)	67 (13.4)	0.334
Hybrid surgery, n (%)	141 (4.4)	120 (4.4)	21 (4.2)	0.858
Staged embolization + Microsurgery, n (%)	83 (2.6)	67 (2.4)	16 (3.2)	0.325
Embolization + SRS, n (%)	381 (11.8)	312 (11.4)	69 (13.8)	0.123
Microsurgery + SRS, n (%)	45 (1.4)	39 (1.4)	6 (1.2)	0.695
Microsurgery + Embolization + SRS, n (%)	50 (1.5)	41 (1.5)	9 (1.8)	0.612
Follow-up duration (years), median, IQR	5.6 (3.4, 7.9)	6.3 (4.4, 8.3)	2.4 (1.8, 3.3)	< 0.001^*^
Lost follow-up, n (%)	253 (7.8)	204 (7.4)	49 (9.8)	0.071
Patients who have completed 3 years follow-up, n (%)	2372 (79.4)	2181 (86.0)	191 (42.4)	< 0.001^*^

*IQR* Interquartile Range, *N* Denominator, *n* numerator, *MATCH* Registry of Multimodality treatment for brain ArTeriovenous malformation in mainland China, *mRS* Modified Rankin Scale, *SD* Standard Deviation, *SRS* Stereotactic Radiosurgery

^*^Statistical significance (*p* < 0.05)

Baseline imaging characteristics of the included AVMs are presented in Table 4. Among them, 87.8% were supratentorial, and the median maximum diameter was 2.9 cm, 55.4% involved eloquent area. Due to poor image quality or absence of requisite sequences, the imaging characteristic data of some patients were missing (less than 2%). Overall, the median Spetzler-Martin and Lawton-Young grades were 3 and 5, respectively. There were no statistically significant differences in imaging characteristics between single-discipline and multidiscipline cohorts.

**Table 4:** Baseline imaging characteristics of the participants in the MATCH study

Characteristics	All patients	Single-disciplinary cohort (2011.08–2021.04)	Multidisciplinary cohort (2018.06–2021.04)	*P* value
No. of patients	*N* = 3241	*N* = 2741	*N* = 500	
Location, supratentorial, n (%)	2844 (87.8)	2417 (88.2)	427 (85.4)	0.081
Maximum diameter (cm), median, IQR	2.9 (2.0, 4.3)	2.9 (2.0, 4.3)	3.0 (2.1, 4.3)	0.468
Eloquence, n (%)				0.149
Yes	1794 (55.4)	1532 (55.9)	262 (52.4)	
No	1447 (44.6)	1209 (44.1)	238 (47.6)	
Feeding artery dilation, n (%)				0.526
Yes	1499 (46.3)	1275 (46.9)	224 (45.3)	
No	1714 (52.9)	1444 (53.1)	270 (54.7)	
Missing	28 (0.9)	22 (0.8)	6 (1.2)	
No. of feeding artery, median, IQR	2 (1, 3)	2 (1, 3)	2 (1, 3)	0.547
Missing	29 (0.9)	22 (0.8)	7 (1.4)	
Watershed AVM, n (%)				0.984
Yes	886 (27.3)	749 (27.5)	137 (27.5)	
No	2339 (72.2)	1978 (72.5)	361 (72.5)	
Missing	16 (0.5)	14 (0.5)	2 (0.4)	
Flow-related aneurysm, n (%)				0.830
Yes	539 (16.6)	458 (16.9)	81 (16.5)	
No	2670 (82.4)	2259 (83.1)	411 (83.5)	
Missing	32 (1.0)	24 (0.9)	8 (1.6)	
Diffuse nidus, n (%)				0.755
Yes	1158 (35.7)	975 (35.9)	183 (36.6)	
No	2060 (63.6)	1743 (64.1)	317 (63.4)	
Missing	23 (0.7)	23 (0.8)	0 (0.0)	
Deep venous drainage, n (%)				0.159
Yes	1258 (38.8)	1078 (39.6)	180 (36.3)	
No	1957 (60.4)	1641 (60.4)	316 (63.7)	
Missing	26 (0.8)	22 (0.8)	4 (0.8)	
No. of draining vein, median, IQR	1 (1, 2)	1 (1, 2)	1 (1, 2)	0.103
Missing	29 (0.9)	22 (0.8)	7 (1.4)	
Drainage vein stenosis, n (%)				0.622
Yes	506 (15.6)	432 (15.9)	74 (15.0)	
No	2706 (83.5)	2287 (84.1)	419 (85.0)	
Missing	29 (0.9)	22 (0.8)	7 (1.4)	
Venous pouches, n (%)				0.560
Yes	506 (15.6)	424 (15.6)	82 (16.6)	
No	2706 (83.5)	2295 (84.4)	411 (83.4)	
Missing	29 (0.9)	22 (0.8)	7 (1.4)	
Spetzler-Martin Grade, median, IQR	3 (2, 3)	3 (2, 3)	3 (2, 3)	0.348
Missing	26 (0.8)	22 (0.8)	4 (0.8)	
Lawton-Young Grade, median, IQR	5 (4, 6)	5 (4, 6)	5 (4, 6)	0.788
Missing	27 (0.8)	23 (0.8)	4 (0.8)	

*AVM* Arteriovenous Malformation, *IQR* Interquartile Range, *N* Denominator, *n* numerator, *MATCH* Registry of Multimodality treatment for brain ArTeriovenous malformation in mainland China, *mRS* Modified Rankin Scale, *SD* Standard Deviation

## Discussion

The MATCH study was the first national prospective registry for AVMs in mainland China. This study is dedicated to exploring the current status of AVM treatment in mainland China (covering most provinces, municipalities, and autonomous regions) and identifying the natural history of AVMs in the Asian population. In addition, this study wants to investigate traditional and emerging hemorrhagic predictors, and explore the superiority of the multidisciplinary assessment in improving the long-term outcomes. On this basis, we will explore the optimal individualized treatment strategies.

Ruptured AVMs should be actively intervened because of the high re-rupture risk [8, 19]. However, the teatment options for unruptured AVMs are currently controversial [20]. ARUBA was the first trial to compare the effects of medical therapy and intervention. During the 33-month follow-up, the medical therapy proved superior in preventing stroke and death [11]. However, the results were strongly criticized regarding several aspects of study design, progression, analysis, and conclusions [21]. Many neurosurgical centers even reported contrary results [22, 23]. However, in 2020, the ARUBA research team reached the final results of ARUBA after long-term follow-up (50.4 months) of 226 patients: medical management alone remained superior to intervention for the prevention of death or symptomatic stroke in unruptured AVMs [12]. After that, a study from the National Inpatient Sample in the USA suggested that the ARUBA trial has influenced the treatment pattern of unruptured AVM, with the overall treatment rate of unruptured AVM decreasing [24]. However, it is worth considering whether medical therapy can benefit the clinically assessed AVMs at high rupture risk (such as combined with exclusive deep venous drainage, and flow-related aneurysm). Therefore, exploring an individualized and optimal treatment protocol based on rupture risk assessment and long-term neurological prognosis prediction is necessary.

Three AVM-related registration trials are cited in PubMed and recruiting currently. The first study, the Treatment of Brain AVMs (TOBAS), started recruiting in 2015 [25]. The general objective is to offer a care trial context for the management of ruptured and unruptured AVMs. In 2017, the TOBAS research team reported encouraging single-center recruitment rates and 61 patients were enrolled in the registry study [26]. The second study, the Multicenter AVM Research Study (MARS) study, an international observational cohort study, concentrated on the long-term outcomes and hemorrhagic predictors in unruptured AVMs [27]. In 2014, the MARS investigators reported that hemorrhagic presentation and increasing age were independent predictors of AVM rupture. It should be noted that MARS will perform causal analysis between the randomized data (ARUBA) and observational data (MARS). The third study, the New ASSessment of cerebral Arteriovenous Malformation yet Unruptured (NASSAU) study, a retrospective study comprising multicenter data from 1351 ARUBA-eligible patients who underwent Gamma Knife Surgery (GKS) [28]. They suggested that the period to break even for morbidity and mortality of AVMs treated with GKS compared to untreated patients in ARUBA was found to be 5 years. The NASSAU is a well-conceived study and reveals the severe limitations of ARUBA.

Exception for the TOBAS study, most of the aforementioned registry studies only focus on unruptured AVMs, and the current disclosed data in the TOBAS study are not encouraging in terms of the recruitment of participating centers and patients. Besides, the optimal intervention strategy for ruptured AVMs remains elusive, especially in patients with similar interventional indications or complex angioarchitecture. The MATCH study can better address this gap as the nationwide prospective registry that can provide adequate samples for prognostic analysis of each treatment strategy and can support the establishment of the prognostic prediction model. Of course, the MATCH study will also explore optimal treatment strategies for unruptured AVMs and develop predictive models that can accurately predict long-term outcomes, and compare the predictive performance with previous models [29].

Another strength of the MATCH study was that novel hemodynamic parameter are introduced and will be used to create rupture prediction models with better predictive performance than previous models [4]. The characteristic angioarchitecture is caused by specific hemodynamics. Color-coded DSA is a breakthrough research direction in recent years for AVM hemodynamic studies [16, 17, 30], and the MATCH study will explore it in depth. The MATCH study will use the NCC study to explore and validate hemorrhagic predictors and build rupture prediction models based on this, which is expected to provide precise prediction of future cerebrovascular events in AVMs. Besides, the MATCH study will explore the superiority of multidisciplinary comprehensive assessment, promote the formulation of individualized treatment protocols conducted by multidisciplinary teams, and effectively promote precision medicine.

There are several limitations of MATCH. First, despite being represented as a whole, potential selection bias of patients was unavoidable since the selected neurosurgical center in this study mostly represented the hospitals with more medical resources and expertise than the low-level hospitals. Second, the MATCH study did not involve the uniform collection and analysis of biological samples from multiple centers due to research funding constraints. Third, the Multidisciplinary Professional Committee of MATCH developed the multidisciplinary standardized treatment protocol to reduce workload. However, the protocol contains overlapping indications between different treatment strategies, which would lead to the deviation of grouping. The multidisciplinary team will decide whether to classify them as experimental or control groups at the regular meetings. Finally, the experimental group has significant less samples than the control group, but with the integration and promotion of the multidisciplinary model, the difference in sample size will be eliminated.

## Conclusion

The MATCH study is a large-sample nationwide prospective registry to investigate multimodality management strategy for AVMs. Data from this registry may also support individualized rupture risk assessment and optimal management strategies selection.

## Supplementary Information

**Additional file 1:** **Appendix S1. **The complete list of MATCH members and sites.

## Data Availability

All data relevant to the study are included in the article or uploaded as supplementary information.

## Abbreviations

ARUBA: A Randomized Trial of Unruptured Brain Arteriovenous Malformations AVMArteriovenous malformation
DTI: Diffusion tensor imaging
DSA: Digital Subtraction Angiography
EDC: Electronic Data Capture
FWHM: Full width at half maximum
GKS: Gamma Knife Surgery
IQR: Interquartile range
IPTW: Inverse probability of treatment weighted
mRS: Modified Rankin Scale
MARS: Multicenter AVM Research Study
MATCH: Multimodality treatment for brain ArTeriovenous malformation in mainland CHina
MRI: Magnetic resonance imaging
NCC: Nested case-controlled
PSM: Propensity score matching
NASSAU AVMs: New ASSessment of cerebral Arteriovenous Malformation yet Unruptured
ROCs: Receiver operating curves
SD: Standard deviation
SRS: Stereotactic radiosurgery
SWI: Susceptibility-weighted imaging
3D T1w MPRAGE: Three-dimensional T1-weighted Magnetization Prepared Rapid Acquisition Gradient Echo
3D-TOF MRA: Three-dimensional time-of-flight MR angiography
TTP: Time to peak
TRV: Trans-nidal relative velocity
TOBAS: Treatment of Brain AVMs
2D T2w: Two-dimensional T2-weighted
