# Supplementary material for: Multimodality treatment for brain arteriovenous malformation in Mainland China: design, rationale, and baseline patient characteristics of a nationwide multicenter prospective registry
Source: Chin Neurosurg J. 2022 Oct 17;8:33. doi: 10.1186/s41016-022-00296-y (PMC9575306; doi:10.1186/s41016-022-00296-y)
Supplement: Supplementary file 1 — Additional file 1: Appendix S1. The complete list of MATCH members and sites. [file 41016_2022_296_MOESM1_ESM.docx]

**Appendix S1. The complete list of MATCH members and sites.**

Beijing Tiantan Hospital, Capital Medical University, Beijing, China: Yuanli Zhao, MD; Peking University International Hospital, Peking University, Beijing, China: Yukun Zhang, MD; West China Hospital, Sichuan university, Sichuan, China: Rui Tian, MD; First Affiliated Hospital of Nanchang University, Jiangxi, China: Jing Jin, MD; First Affiliated Hospital of Harbin Medical University, Heilongjiang, China: Pei Wu, MD; Sichuan Provincial People's Hospital, Sichuan, China: Ling Liu, MD; The First Hospital of Jilin University, Jilin, China: Tianyi Liu, MD; Air Force Military Medical University Tangdu Hospital, Shaanxi, China: Wei Fang, MD; Shanxi Provincial People's Hospital, Shanxi Medical University, Shanxi, China: Xudong Hao, MD; The Second Hospital of Shandong University, Shandong, China: Yuefei Fan, MD; The First Affiliated Hospital of Zhengzhou University, Henan, China: Xinpu Chen, MD; The First Affiliated Hospital of Xinjiang Medical University, Xinjiang, China: Maimaiti Liaisha, MD; The General Hospital of Central Theater Command, Hubei, China: Yueyuan Zhao, MD; The Affiliated Hospital Of Guizhou Medical University, Guizhou, China: Han Peng, MD; The First Affiliated Hospital of Guangxi Medical University, Guangxi, China: Shengyong Wang, MD; Tianjin Medical University Second Hospital, Tianjin, China: Dong Liu, MD; The First Affiliated Hospital of Shandong First Medical University, Shandong, China: Lei Zhang, MD; Shengjing Hospital of China Medical University, Liaoning, China: Wei Tang, MD; The First Affiliated Hospital of Xi 'an Jiaotong University, Shaanxi, China: Chunying Ren, MD; The Second Affiliated Hospital of Guangzhou Medical University, Guangzhou, China: Jianfeng Liang, MD; Tianjin Huanhu Hospital, Tianjin, China: Ruiting Zhao, MD; Chifeng City Hospital, Inner Mongolia, China: Xuguang Liang, MD; Hainan People's Hospital, Hainan, China: Renduan Cai, MD; The Second Affiliated Hospital of Kunming Medical University, Yunnan, China: Xiaoli Min, MD; The Second Hospital of Shandong University, Shandong, China: Jun Jiang, MD; The Affiliated Hospital of Qingdao University, Shandong, China: Zhiyong Yan, MD; The First Affiliated Hospital of Shihezi University, Xinjiang, China: Jing Dai, MD; The Second Xiangya Hospital of Central South University, Hunan, China: Zhongzhong Jiang, MD; The Second Affiliated Hospital of Soochow University, Jiangsu, China: Qing Zhu, MD; Hospital of Xinjiang Production and Construction Corps, Xinjiang, China: Li Yu, MD; General Hospital of Ningxia Medical University, Ningxia, China: Wei Wang, MD; The Second Hospital of Lanzhou University, Lanzhou, China: Shouyuan Sun, MD; The First Affiliated Hospital of Anhui Medical University, Anhui, China: Jing Luo, MD; The First Affiliated Hospital of Zhejiang University, Zhejiang, China: Qingsheng Xu, MD; Qilu Hospital of Shandong University, Shandong, China: Yunyan Wang, MD; Affiliated Hospital of Jining Medical College, Shandong, China: Wanju Zhao, MD; The First Affiliated Hospital of Zhejiang University, Zhejiang, China: Min Yan, MD; The First Affiliated Hospital of Chongqing Medical University, Chongqing, China: Hongbin Liu, MD; The First Affiliated Hospital of Fujian Medical University, Fujian, China: Fuxin Lin, MD; Shandong Provincial Hospital, Shandong, China: Bin Feng, MD; The Affiliated Hospital of Xuzhou Medical University, Jiangsu, China: Xiaofeng Chao, MD; Affiliated Hospital of Binzhou Medical College, Shandong, China: Yonggang Ma, MD; Henan Provincial People's Hospital, Henan, China: Junshi Shao, MD; The Affiliated Nanjing Drum Tower Hospital of Nanjing University Medical School, Jiangsu, China: Zhiyong Shi, MD; Renji Hospital affiliated to Shanghai Jiaotong University, Shanghai, China: Jianpo Zhou, MD; Yancheng Third People's Hospital, Jiangsu, China: Ya Xue, MD; Binhai Hospital affiliated to Tianjin Medical University, Tianjin, China: Liang Wang, MD; Tongren Hospital, Capital Medical University, Beijing, China: Yong Xu, MD; Beijing Children's Hospital, Capital Medical University, Beijing, China: Rutao Luo, MD; Beijing Jishuitan Hospital, Beijing, China: Longqi Liu, MD; Chaoyang Hospital, Capital Medical University, Beijing, China: Yibo Geng, MD; Beijing Tsinghua Changgung Hospital affiliated to Tsinghua University, Beijing, China: Yi Guo, MD;
